# Augmenting the Antitumor Efficacy of Natural Killer Cells via SynNotch Receptor Engineering for Targeted IL-12 Secretion

**DOI:** 10.3390/cimb46040183

**Published:** 2024-03-28

**Authors:** Ali Ahmadnia, Saeed Mohammadi, Ahad Yamchi, Mohamad Reza Kalani, Touraj Farazmandfar, Ayyoub Khosravi, Ali Memarian

**Affiliations:** 1 Department of Molecular Medicine, Faculty of Advanced Medical Technologies, Golestan University of Medical Sciences, Gorgan P.O. Box 665, Iran; 2 Golestan Research Center of Gastroenterology and Hepatology, Golestan University of Medical Sciences, Gorgan P.O. Box 665, Iran; 3 Department of Biotechnology, Gorgan University of Agricultural Sciences and Natural Resources, Gorgan P.O. Box 386, Iran; 4 Medical Cellular and Molecular Research Center, Golestan University of Medical Sciences, Gorgan P.O. Box 665, Iran; 5 Stem Cell Research Center, Golestan University of Medical Sciences, Gorgan P.O. Box 665, Iran; 6 Department of Medical Immunology, School of Medicine, Golestan University of Medical Sciences, Gorgan P.O. Box 665, Iran

**Keywords:** NK cell, synNotch receptor, IL-12, IFNγ, cytotoxicity, breast cancer, cancer immunotherapy

## Abstract

Natural killer (NK) cells are crucial components of innate immunity, known for their potent tumor surveillance abilities. Chimeric antigen receptors (CARs) have shown promise in cancer targeting, but optimizing CAR designs for NK cell functionality remains challenging. CAR-NK cells have gained attention for their potential to reduce side effects and enable scalable production in cancer immunotherapy. This study aimed to enhance NK cell anti-tumor activity by incorporating PD1-synthetic Notch (synNotch) receptors. A chimeric receptor was designed using UniProt database sequences, and 3D structure models were generated for optimization. Lentiviral transduction was used to introduce PD1-Syn receptors into NK cells. The expression of PD1-Syn receptors on NK cell surfaces was assessed. Engineered NK cells were co-cultured with PDL1+ breast cancer cells to evaluate their cytotoxic activity and ability to produce interleukin-12 (IL-12) and interferon-gamma (IFNγ) upon interaction with the target cells. This study successfully expressed the PD1-Syn receptors on NK cells. CAR-NK cells secreted IL-12 and exhibited target-dependent IFNγ production when engaging PDL1+ cells. Their cytotoxic activity was significantly enhanced in a target-dependent manner. This study demonstrates the potential of synNotch receptor-engineered NK cells in enhancing anti-tumor responses, especially in breast cancer cases with high PDL1 expression.

## 1. Introduction

Natural killer (NK) cells are critical components of innate immunity that play a critical role in tumor immunosurveillance. Unlike other immune cells that require prior sensitization, NK cells can rapidly identify and eliminate malignant cells using a diverse array of genome-encoded surface receptors, such as killer immunoglobulin-like receptors (KIRs) and natural cytotoxicity receptors (NCRs) [1]. Similar to cytotoxic T lymphocytes, NK cells have the ability to produce and release cytotoxic granules containing perforin and granzymes, which enables direct tumor cell lysis upon activation [2,3]. Additionally, NK cells modulate adaptive immune responses via cytokine and chemokine production, such as tumor necrosis factor-alpha (TNFα), interferon-gamma (IFNγ), interleukin-10 (IL-10), and granulocyte-macrophage colony-stimulating factor (GM-CSF) [4,5]. These properties make NK cells a focus of cancer immunotherapy research [6,7].

Chimeric antigen receptors (CARs) offer a promising avenue to enhance cancer cell targeting. CAR-NK cells, distinct from T-cell-based therapies, demonstrate reduced adverse effects and serve as an “off-the-shelf” product, meeting the clinical need for large-scale production in cancer immunotherapy [8,9,10,11]. However, CAR design optimization for NK cell function remains challenging [12].

Synthetic Notch (synNotch) receptors have emerged as versatile tools for tailoring cellular responses to user-specific antigens, allowing precise control over therapeutic interventions by triggering the activation and release of intracellular transcription factors [13]. Comprising key components, including a regulatory domain from the Notch receptor, a synthetic intracellular transcriptional domain, and an extracellular recognition domain resembling a single-chain antibody, synNotch receptors facilitate the translocation of the transcriptional domain and the activation of target genes in the nucleus. The distinctive design of the synNotch receptors enables the precisely targeted delivery of therapeutic payloads, such as cytokines, to tumor sites [13,14,15].

IL-12, a proinflammatory cytokine in both innate and adaptive immunity, boosts antitumor responses by activating tumor-infiltrating lymphocytes (TILs), inhibiting Treg-mediated regulation, and promoting NK cell migration [16,17]. Yet, systemic IL-12 administration as a therapeutic mediator faces toxicity limitations [18,19], necessitating localized production at the tumor sites.

The programmed death-1 (PD1)/programmed death ligand-1 (PDL1) pathway influences lymphocyte tolerance and cancer progression [20]. PDL1 expression correlates with suppressed NK cell responses and tumor aggressiveness. Remarkable success has been achieved in treating various cancer types using immunotherapy targeting the PD1/PDL1 axis [21,22,23,24], which underscores the role of the PD1/PDL1 axis in NK cell responses [25,26].

Due to the high level of PDL1 expression in several tumors, in this study, we developed synNotch receptors to create PD1-Syn-IL-12 NK cells, allowing precise IL-12 expression, followed by PDL1 ligation. This research explores the potential of IL-12 production by PD1-Syn-IL-12 NK cells in enhancing antitumor therapy. Our goal is to optimize the potential therapeutic outcomes using IL-12 modulation.

## 2. Materials and Methods

### 2.1. Design of SynNotch PD1 Chimeric Receptors

We obtained protein sequences corresponding to the human PD1 extracellular domain (amino acids 21–170) and the human Notch1 extracellular regulatory region, including the extended form, from the Universal Protein Resource (UniProt database, Q15116, PDCD1_HUMAN). Using the NCBI protein BLAST tool, with default parameters for Homo sapiens, we identified suitable templates for protein structure prediction. The recombinant constructs were generated by fusing the PD1 extracellular domain with the basic and extended forms of the human Notch1 extracellular regulatory region.

### 2.2. Modeling of Extracellular Receptor Structure

We utilized the I-TASSER server, recognized as one of the leading computational tools for protein structure prediction (https://zhanglab.ccmb.med.umich.edu/I-TASSER/ (accessed on 15 February 2020)) [27] to generate the 3D structure models of the transmembrane and extracellular domains of PD1, as well as the extracellular region of the synNotch receptors. The server is continuously evolving with the aim of providing the most accurate protein structure and function predictions using state-of-the-art algorithms. To enhance the accuracy, we implemented various strategies for model refinement and optimization. Specifically, for the PD1 receptor, we explored conformational dynamics by inserting different linkers between the two parts of the extracellular domain. Subsequently, the models underwent further refinement using advanced algorithms and simulations by the server. To validate the accuracy of the predicted models, we compared them with experimentally determined structures using the PDB RCSB Structure Pairwise Alignment Tool (https://doi.org/10.2210/pdb3BIK/pdb (accessed on 15 February 2020)) to assess the structural similarities and deviations.

### 2.3. Plasmid Production

The PD1 synNotch receptor was created by fusing two distinct domains, comprising the extracellular domain of human PD1 and the intracellular domain of the Notch core. This integration combined the binding specificity of PD1 with the transcriptional activation capabilities of the Notch pathway. This design enabled the receptor to identify specific ligands, initiating downstream signaling events that activated the target genes. Additionally, the incorporation of the Gal4VP64 domain further enhanced the controllability of the receptor, as it can be induced by the Gal4-inducible promoter.

To ensure the efficient delivery and expression of the PD1 synNotch receptor, a lentiviral vector system was utilized. The lentiviral vector, PCDH, provided by Biomatic (Quebec, QC, Canada), was selected for its ability to efficiently transduce the target cells and facilitate the stable integration of the receptor construct into the host genome. The CMV promoter was employed to drive the expression of the receptor construct and facilitate robust and consistent expression levels. The GFP gene, regulated by the CMV promoter, is a marker for transduced NK cells. This allowed for the identification and tracking of the cells that have successfully integrated and expressed the PD1 synNotch receptor construct.

### 2.4. Cell Culture and Cell Lines

We sourced the human HEK293 (CRL-3216) cell line, the human breast cancer cell lines, MCF7 (HTB-22) and MDA-MB231 (HTB-26), and the human NK92 (CRL-2407) cells from the National Cell Bank of Iran (NCBI, Tehran, Iran). The NK92 cells were cultured in a medium consisting of heat-inactivated 12.5% human AB serum, 12.5% heat-inactivated fetal bovine serum (FBS; Gibco, Billings, MT, USA), and 200 U/mL recombinant human IL-2 [28]. All the other cell lines were cultured in DMEM or RPMI-1640 supplemented with 10% FBS, 100 IU/mL (Catalog no 16000044) of penicillin, and streptomycin (Catalog number: 15140122) (all from Gibco). Peripheral blood mononuclear cells (PBMCs) were isolated from healthy human donors using Ficoll-Hypaque (Baharafshan, Tehran, Iran) gradient centrifugation. The NK cells from the PBMCs were separated using the Human NK Cell negative selection Kit (Miltenyi Biotec, Bergisch Gladbach, Germany, Catalog no 130-092-657). The isolated NK cells were cultured with 25 ng/mL IL-15 in an a-MEM medium supplemented with 100 IU/mL IL-2 (Biolegend, San Diego, CA, USA) and 12.5% human autologous serum [29,30].

### 2.5. Production and Transduction of Lentiviruses

The HEK293 cells were transfected with the lentiviral constructs, pCDH-CMV PSPAX and pMD2.G, using the PEI (polyethyleneimine) reagent, as previously described [31,32]. The lentiviral constructs, pCDH-CMV PSPAX and pMD2.G, were mixed with the PEI reagent in the Opti-MEM medium and incubated at room temperature for 15 min to allow complex formation. The lentiviral–PEI complexes were then added dropwise to the HEK293 cells, followed by incubation for 6–8 h at 37 °C in a CO_2_ incubator. Subsequently, the lentiviral particles were isolated and concentrated by subjecting the supernatant containing the viral particles to a two-hour ultracentrifugation at 42,000× *g*. The supernatant was carefully aspirated, and the viral pellet was re-suspended in an appropriate volume of sterile culture medium to achieve the desired concentration of the lentiviral particles [33]. The NK92 and primary NK cells were transduced following one week of expansion. A day before transduction, RetroNectin (Takara, Shiga, Japan), Catalog No. 50-444-031) was applied to a 12-well plate as a coating. On the subsequent day, the concentrated vectors with specified multiplicities of infection (MOI) were introduced to the coated plates and incubated at 37 °C for 4 h. The NK cells were then seeded into these wells along with the IL-2-supplemented medium. The plates were centrifuged at 1000× *g* for 1 h and incubated overnight at 37 °C. The following day, the IL-2-supplemented medium was added to each well. The transduction efficiency was evaluated on day 3 for NK92 and the freshly isolated NK cells after the transduction process.

### 2.6. Assessment of Chimeric Receptor Functionality: Cytotoxicity and Cytokines Assays

Co-cultures of transduced NK cells and tumor cells were established in triplicate in 96-well plates to assess the chimeric receptor activity. After a 24-h incubation period, we collected the supernatants for the cytotoxicity and cytokine assays. The transduced NK cell cytotoxicity was assessed at various effector-to-target ratios (E:T ratios), including 1:2, 1:1, 2:1, and 4:1, using the LDH release test and the LDH Cytotoxicity Assay Kit (Kiazist, Hamadan, Iran), following the manufacturer’s instructions. Additionally, we determined the concentrations of IFNγ and IL-12 in the culture supernatant at E:T ratios of 1:1, 1:3, and 1:5 using the ELISA kits (ELISA MAX, Biolegend, USA Catalog No. 50-169-392), following the manufacturer’s guidelines.

### 2.7. Flow Cytometry Analysis

To assess the expression of synNotch-PD1-CAR on the transfected NK92 and primary NK cells, as well as PDL1 on the cell lines, we employed flow cytometry as previously described [34]. The cells were stained with PE-conjugated anti-human PD1 and FITC-conjugated anti-human PDL1 antibodies (Biolegend, USA). After 1 h incubation at 4 degrees Celsius, the cells were washed and resuspended in PBS containing 0.1% NaN3 and 0.5% paraformaldehyde for a subsequent analysis using the BD Accuri C6 flow cytometer (Becton, Dickinson and Company, Franklin Lakes, NJ, USA).

### 2.8. Statistical Analysis and Evaluation

We performed a statistical analysis utilizing GraphPad Prism version 8.0. We employed unpaired two-way analysis of variance (ANOVA) or the Kruskal–Wallis non-parametric test to compare the means between the different groups. Our experiments were conducted with both biological and experimental replicates, with each assay performed in triplicate. We considered the statistical significance at *p* < 0.05 and regarded *p* < 0.001 as indicative of extreme significance.

## 3. Results

### 3.1. Designing and Validating the synNotch PD1 Vector

**Figure 1:**
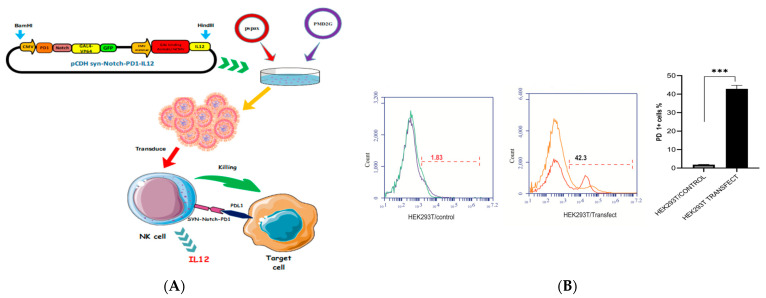
Designing and validating the synNotch PD1 vector: (**A**) The schematic diagram of the synNotch-PD1-CAR transgene illustrates the synNotch and GAL4 circuits, enabling the cellular response activation with the corresponding bright field images. (**B**) Using flow cytometry, the percentage of HEK293T cells transfected with the synNotch-PD1-CAR construct was determined, using GFP expression serving as a cell marker (almost 42% GFP-positive cells). Insights into the successful transfection after 48 h were obtained by comparing the resulting fluorescent images. Each experiment was repeated at least twice with similar results. Data are presented as the mean ± SEM, and significant differences between the groups were measured using one-way analysis of variance (ANOVA). *** *p* < 0.001. Only the significant differences are indicated.

Figure 1A illustrates the architecture of the synNotch and GAL4 circuits. To enable a cellular response, a reporter construct was created with a responsive promoter. Upon activation by the synNotch-induced transcription factor, this led to the expression of the IL-12 gene. We utilized the extracellular domain of PD1 to develop a functional synNotch receptor with the ability to bind the PDL1 ligand. This synNotch receptor was engineered by fusing the Notch core region with an artificial transcription factor, known as Gal4VP64. Within the reporter construct, the green fluorescent protein (GFP) was placed under the control of a Gal4VP64 responsive promoter. Consequently, when the synNotch-PD1-CAR receptor became activated, the Gal4VP64 was released and translocated into the nucleus. Once in the nucleus, Gal4VP64 took charge of regulating the expression of the reporter gene. The transfected HEK293 cells were examined using fluorescent microscopy to confirm the transfection by GFP expression. The proportion of the HEK293T cell line successfully transfected with the synNotch-PD1-CAR construct was determined using flow cytometry, where PD1 expression served as a surface marker. By analyzing the fluorescence signals from the cells, we quantified that nearly 42% of the cells underwent successful transfection (Figure 1B).

### 3.2. Protein Modeling

We employed three different methods to predict the structure of the chimeric proteins. Two forms of the synNotch-PD1 structure models were successfully generated using both the Robetta and I-TASSER servers [35], which produced five top-predicted models based on their scoring algorithms. Additionally, the modeler algorithm generated five models, and the best among them was selected as the synNotch protein model [36].

For validation and quality assessment purposes, the best models from each method were chosen. The predicted protein structures underwent validation using both the QMEAN and Prosa servers, considering their Ramachandran total G-Factor [37]. The ANOLEA server was utilized to provide a visual representation of the results, illustrating the number of amino acids residing in energetically favorable environments with acceptable QMEAN scores [38].

Furthermore, we subjected the generated models to validation studies using the PROCHECK program, which assessed the Ramachandran plot and calculated the overall G-Factor for each model. The quality factor of the models was represented by the ERRAT plots [39,40]. The data unequivocally indicated that the model generated by the Robetta server exhibited the highest quality. Consequently, we conducted further analyses and investigations using the model predicted by this server.

**Figure 2:**
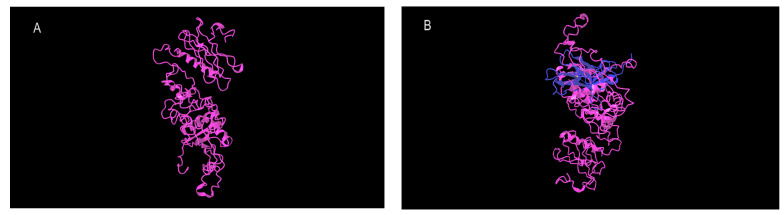
Prediction and validation of synNotch-PD1 structure models: Three methods were employed to predict the structures of the chimeric proteins, resulting in the generation of two synNotch-PD1 models. Validation was conducted using QMEAN, Prosa, ANOLEA, and PROCHECK, assessing the energetically favorable environments, Ramachandran plots, and overall G-Factors. The model produced by the Robetta server exhibited the highest quality, warranting further analysis. The chimeric receptor combined the Notch1 receptor and the PD1 extracellular sequences with the linkers. The best model featured the GGGGSGGGGSGGGGS linker, closely resembling the natural structure of the PD1 extracellular domain. (**A**) 3D structure of synNotch-PD1 with (GGGGS)3 linker; (**B**) Structure alignment with native PD-1 (blue) by TM-align server.

For the modeling of the chimeric receptor, the extracellular sequence of the Notch1 receptor and the extracellular domain of PD1 were used. These two fragments were interconnected using several linkers. The best model, with the highest score, was associated with the linker GGGGSGGGGSGGGGS, which exhibited the highest similarity to the natural structure of the extracellular domain of PD1 (Figure 2).

### 3.3. Expression of PDL1 Varies between Different Breast Cancer Cell Lines

**Figure 3:**
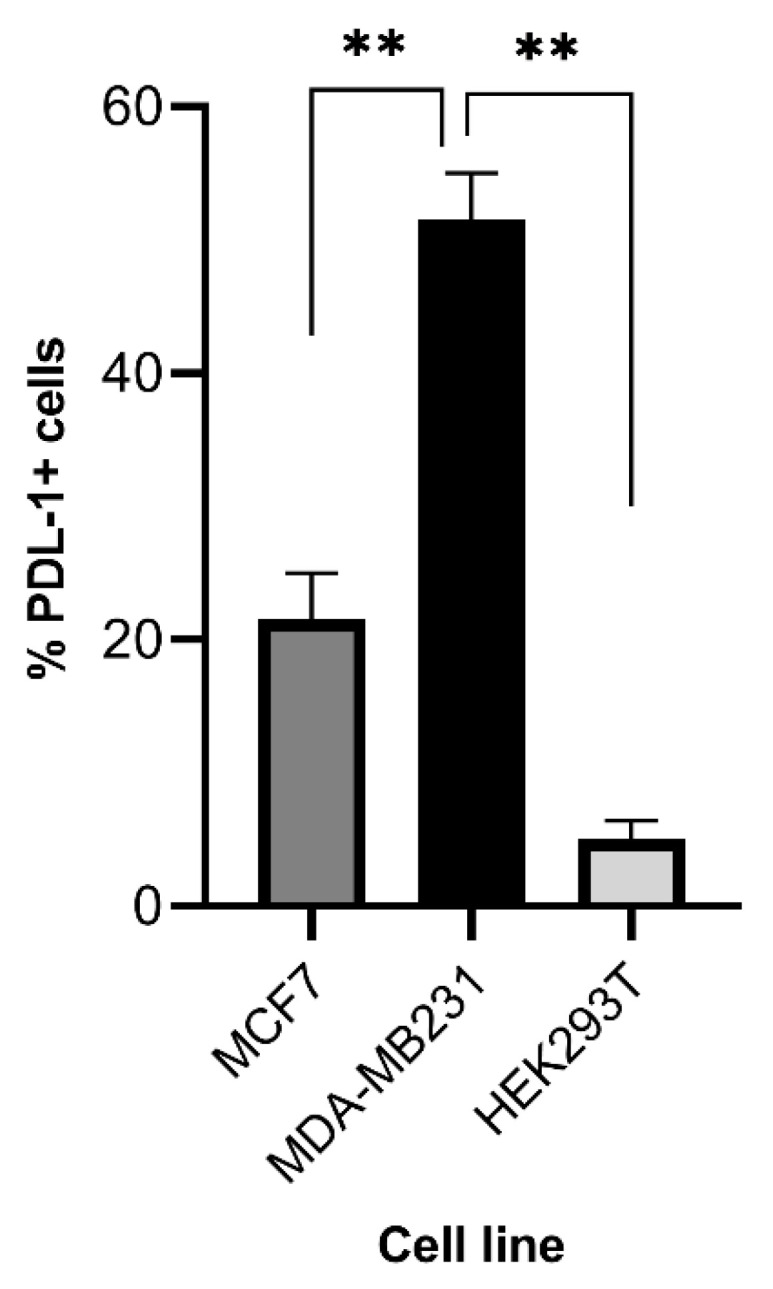
The expression of programmed death-ligand 1 (PDL1) demonstrates variation across different breast cancer cell lines: PDL1 expression was analyzed in the MDA-MB231, MCF-7, and HEK 293T cell lines using flow cytometry (data represented in a histogram chart.). MDA-MB231 showed the highest PDL1 expression, while HEK 293T exhibited low expression and served as a control. The experiments were performed in triplicate. Data are presented as the mean ± SEM, and significant differences between the groups were measured using one-way analysis of variance (ANOVA). ** *p* < 0.01. Only the significant differences are indicated.

PDL1 expression was assessed in embryonic kidney cancer cell lines (HEK 293T) and two human breast cancer cell lines (MCF-7 and MDA-MB231) using flow cytometry. The two human breast cancer cell lines exhibited relatively higher expression of PDL1, with MDA-MB231 displaying the highest level of PDL1 expression. In contrast, the HEK293T cell line demonstrated low expression of PDL1 and served as a control in the study (Figure 3).

### 3.4. Lentiviral Vector Synthesis and Evaluation

**Figure 4:**
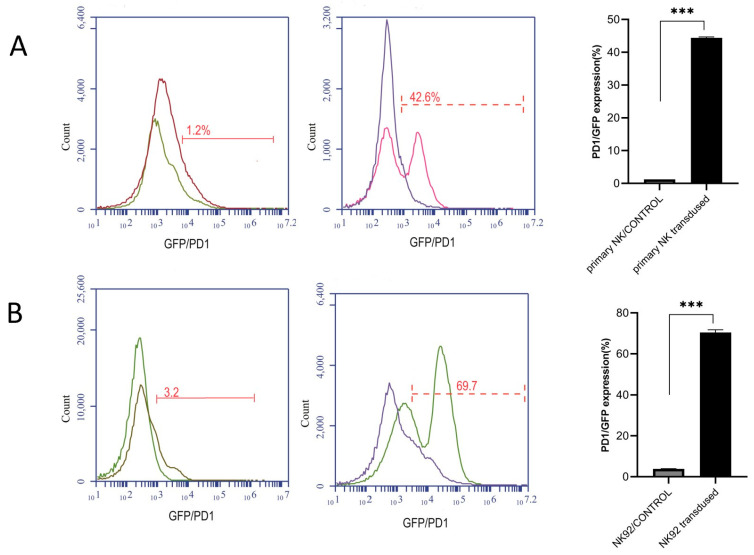
Lentiviral vector synthesis and transduction evaluation were quantified and compared to their non-transduced counterparts using flow cytometry. A flow cytometric analysis of GFP expression was conducted in the primary NK cells and the NK92 cell line against the control group. We further assessed PD1-specific synNotch expression on the primary NK cells and the NK92 cell line using flow cytometry. CAR expression on NK cells was detected utilizing a biotin-conjugated anti-human Fab antibody, followed by PE-conjugated streptomycin. Remarkably, almost 43% of the transduced primary NK cells (**A**) and 70% of the transduced NK92 cells (**B**) effectively exhibited PD1-specific synNotch expression on their surface, in contrast to the control group. Each experiment was repeated at least three times. Data are presented as the mean ± standard deviation (SD), and significant differences between the groups were measured using one-way analysis of variance (ANOVA). *** *p* < 0.001. Only significant differences are indicated.

The lentiviral expression vectors for the GAL4-responsive promoter and the PDL1-specific synNotch receptor are depicted in Figure 4. To achieve persistent expression of the PD1synNotch receptor and enable a proper transcriptional response, the NK cells were transduced using a lentivirus carrying the “PD1-Syn-GFP-NK” construct.

This lentiviral vector combined the synNotch-PD1-CAR with GFP, resulting in the production of GFP under the transcriptional regulation of the CMV promoter (Figure 4A,B). Utilizing flow cytometry, we meticulously quantified and compared the transduced NK cells with their non-transduced counterparts. This precise analysis enabled us to ascertain that nearly 70% of the NK92 cells and 43% of the primary NK cells had effectively integrated GFP expression and the PD1-specific synNotch into their cellular composition.

### 3.5. Interplay between PDL1 Expression, IL-12, and IFNγ Production in PD1-Syn-IL-12-NK92 Cells

**Figure 5:**
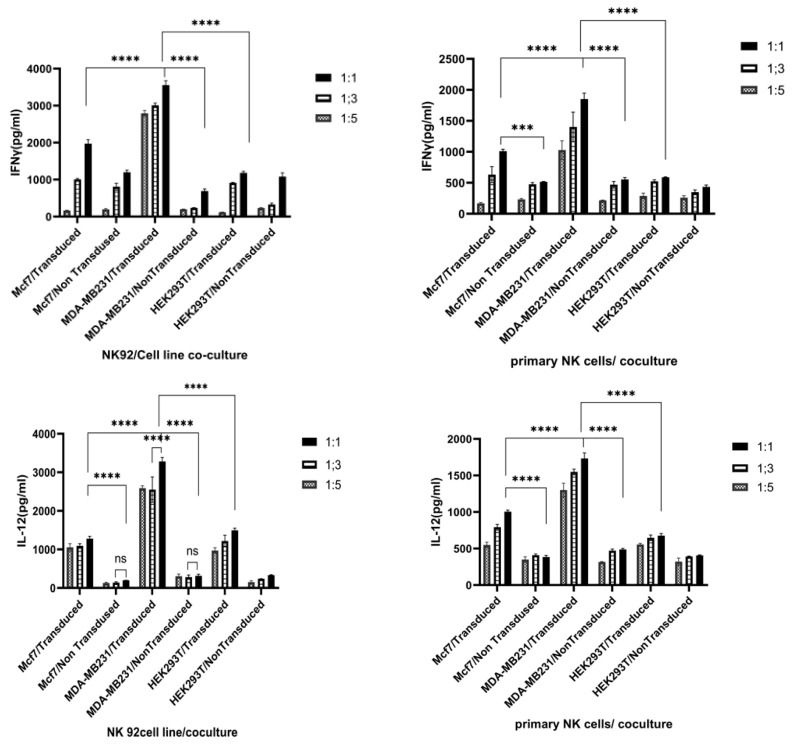
Quantification of IFNγ and IL-12 production following 24-h co-culture of the human primary NK cells and NK92 cell line with PDL1-positive breast cancer target cells by ELISA: The data represent three independent biological replicates of IFNγ and IL-12 levels in three different ratios of effector-to-target cells (E: T ratios). Error bars are the mean ± SD from the three separate experiments. ns: not significant; *** *p* < 0.001, **** *p* < 0.0001.

Following lentiviral transduction, we conducted a comparative analysis of IL-12 expression between the breast cancer cell lines and the un-transduced PD1-Syn-IL-12-NK92 cells. This analysis aimed to investigate the relationship between PDL1 expression on the surface of the cancer cells and IL-12 production. The results demonstrated a correlation between the expression level of tumor cells and the corresponding IL-12 expression by the PD1-Syn-IL-12-NK92 cells. Higher PDL1 expression on the tumor cells was associated with increased IL-12 production by the PD1-Syn-IL-12-NK92 cells. This finding suggests that the PD1-Syn-IL-12-NK92 cells exhibited targeted IL-12 secretion in response to the PDL1-positive tumor cells (Figure 5).

Regarding the cytokine production profiles, our observations indicated that the PD1-Syn-IL-12-NK92 cells significantly increased IFN-γ secretion when co-cultured with the PDL1-positive cells. In contrast, co-culture with the PDL1-negative cells did not induce a similar response.

### 3.6. Enhanced Lysis of PDL1-Positive Breast Cancer Cells by PD1-Syn-IL-12-NK Cells

**Figure 6:**
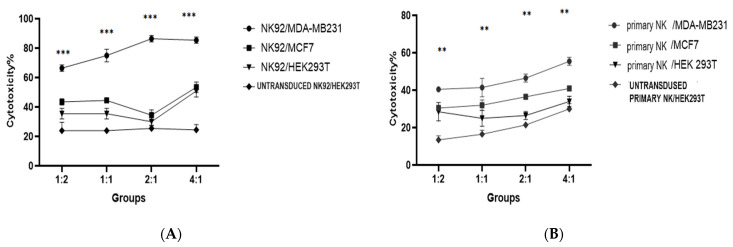
LDH release from PDL1-positive breast cancer target cells: CAR-NK92 and CAR–primary NK cells were tested for their cytotoxic effects on PDL1-positive breast cancer cell lines. The release of LDH from the target cells enhanced the cytolytic activity of these CAR-NK cells against the target cells, which is directly associated with the PDL1 expression. The MDA-MB231 cells induced a significantly high level of cytotoxicity from the CAR-NK92 (**A**) and primary CAR-NK (**B**) cells. The data represent three independent biological replicates in four different ratios of effector-to-target cells (E:T ratios). Error bars are the mean ± standard deviation (SD) from the three separate experiments. ** *p* < 0.01; *** *p* < 0.001.

To assess the impact of the PD1-Syn-IL-12-NK cells on the lysis of the PDL1-positive breast cancer cell lines, an LDH cytotoxicity assay was conducted. The PD1-Syn-IL-12-NK cells were co-incubated with the cells from three different breast cancer cell lines, maintaining NK cell-to-target cell ratios of 1:2, 1:1, 2:1, and 4:1. The objective was to determine the potential of the lysis effects of the PDL1-positive breast cancer cells in vitro. These findings suggest that the higher expression level of PDL1 in the target cells (MDA-MB231) induced higher significant cytotoxicity in both the PD1-Syn-IL-12-NK92 cells and the PD1-Syn-IL-12-NK cells (Figure 6).

## 4. Discussion

The pivotal role of NK cells in tumor immunosurveillance is well-established, supported by extensive studies in murine models and clinical trials involving humans. Diminished NK cell activity has consistently been associated with heightened cancer susceptibility and metastasis, underscoring the significance of comprehending NK cell biology and devising strategies to bolster their efficacy. Understanding NK cell biology and developing interventions to enhance their activity is crucial for advancing effective cancer management and treatment. NK cell-based immunotherapies, owing to their cytotoxic prowess and capacity to identify and eliminate cancer cells, have remained a focal point in cancer research, offering promising avenues for cancer treatment [41,42,43].

This study pioneered the use of synNotch receptors to enhance targeted therapeutic responses in NK cells. Through the innovative application of the synNotch receptors, we have engineered a novel construct, termed “PD1-Syn-IL-12”, which is designed to detect PDL1 and facilitate the expression of IL-12 molecules within the NK92 cells and primary NK cells, affording precise control over IL-12 expression (Figure 1). The NK92 cell line, a well-established NK cell line, has demonstrated both safety and efficacy in allogeneic cell therapy, alongside primary NK cells derived from donors. Clinical responses have been observed in certain patients with cancer treated with CAR-NK92 cells. Clinical trials have further confirmed the safety and effectiveness of NK92 cell infusions [44,45].

Given the multifaceted nature of breast cancer treatment, which typically involves a combination of surgical procedures, radiotherapy, chemotherapy, hormone therapy, and immunotherapy, there is a pressing need for more effective and targeted approaches [46,47]. This study highlights the potential of synNotch receptor-engineered NK cells and their precise IL-12 secretion mechanism as a promising addition to the armamentarium of breast cancer treatments. The complexity of breast cancer necessitates a comprehensive and personalized approach, and these findings suggest that targeted immunotherapeutic strategies hold significant promise in this regard. By harnessing the power of the immune system with precision, we aim to improve treatment outcomes and minimize adverse effects. This research opens new avenues for developing tailored and effective therapies, offering hope for enhanced breast cancer management and potentially benefiting other challenging malignancies. Further in vivo investigations could support and confirm our results in order to improve clinical assessments.

In this investigation, we examined the levels of PDL1 expression in human breast cancer cell lines (MCF-7 and MDA-MB231) and embryonic kidney cancer cell lines (HEK 293T), revealing significant differences in PDL1 expression (Figure 3). Notably, MDA-MB231 exhibited the highest PDL1 levels among the breast cancer cell lines (Figure 3). This variation underscores the importance of considering the individual cell line characteristics when developing immunotherapeutic strategies. In this regard, PDL1 expression on cancer cells is essential for effective engagement with immune cells [48]. Emerging data underscore the regulatory role of various tumor microenvironmental stimuli in PDL1 expression [49,50], which is observed in several cancer types, including renal cell carcinoma, pancreatic cancer, ovarian cancer, gastric cancer, esophageal cancer, and breast cancer, while it remains absent in healthy epithelial tissues [51,52]. Immune checkpoint inhibitors (ICIs), such as pembrolizumab and atezolizumab, which disrupt the PD1–PDL1 interaction and stimulate cytotoxic T lymphocytes (CTLs), have been approved for patients with breast cancer, particularly those with HER2^+^ and triple-negative breast cancer (TNBC) [53,54,55,56]. The elevated level of PDL1 expression serves as a predictive biomarker for ICI therapy effectiveness, often correlating with the presence of TILs [57,58]. These findings highlight the importance of assessing PDL1 expression as a predictive marker for immunotherapy response, particularly in breast cancer. The detection of PDL1 expression in the majority of the MDA-MB231 cells, along with its near absence in the MCF7 and HEK293T cell lines used as controls, underscores the potential utility of targeted immunotherapeutic strategies based on PDL1 expression levels. In previous studies, SynNotch pathways have shown the capability to selectively trigger specific functional responses in various cell types. Conversely, the potent immunostimulatory properties of IL-12, which can activate the innate and adaptive immune systems to promote antitumor immunity, have been well-documented [59]. However, the clinical application of recombinant IL-12 as a therapeutic agent has been hindered by severe side effects, including instances of fatalities [60]. As a result, the systemic administration of therapeutic IL-12 doses in patients with cancer has often been restricted [61,62]. Earlier clinical trials have explored the use of IL-12 gene-transduced tumor cells, fibroblasts, or dendritic cells as delivery vehicles for controlled IL-12 release at tumor sites, but these approaches have displayed limited effectiveness. This highlighted the necessity of combining IL-12 with additional anticancer strategies [63,64,65]. In this context, Hua Jiang and Zonghai Li et al. introduced the SynNotch receptor as a promising method for precise and safe cytokine delivery in tumor treatment. Their research investigated the feasibility of delivering IL-12 through the adoptive transfer of Glypican3-Syn-IL-12-NK92 cells to specific tumor sites. Their findings demonstrated the immune-stimulatory properties of IL-12, as well as the production of IFNγ, while simultaneously inducing PDL1 overexpression. Since PDL1 typically plays an immunosuppressive role, its heightened expression following IL-12 treatment could potentially counteract the anticancer effects of IL-12. This intriguing avenue of research highlights the complex interplay of cytokines and immune responses in the context of cancer therapy [66]. To address this challenge, it is crucial to manage PDL1 expression to preserve the improved immune microenvironment following IL-12 treatment. Consequently, combining PD1/PDL1 blocking therapy with the NK cells modified to secrete IL-12 through the synNotch receptor presents an appealing approach.

Regarding chimeric signaling receptor (CSR) engineering, Prosser et al. introduced a novel and groundbreaking approach involving PD1/CD28 proteins expressed on CTLs. The findings demonstrated that the PD1/CD28 CSR could enhance the phosphorylation of crucial signaling molecules, such as ERK and Akt, thereby profoundly impacting CTL stimulation, proliferation, and cytokine secretion—fundamental processes for an effective immune response against cancer cells [67]. Following this, Ankri et al. highlighted the impact of the PD1/CD28 CSR on cytokine production. The research unveiled that the engineered cells expressing PD1/CD28 exhibited augmented secretion of various cytokines, including critical immune mediators, such as IFNγ and interleukin-2, further substantiating the use of the CSR to modulate immune responses against infections and malignancies [68]. Yangbing Zhao and Carl June’s team also conducted a comprehensive analysis, comparing the structural characteristics of the PD1/CD28 and PD1/4-1BB chimeric-switch receptors. The researchers harnessed the potential of the PD1/CD28 CSR to engineer CAR-T cells targeting mesothelin (MSLN) and prostate stem cell antigen (PSCA). Their investigations revealed that these PD1/CD28 CAR-T cells exhibited significantly enhanced antitumor effects compared with traditional CAR-T cells. Furthermore, these PD1/CD28 CAR-T cells outperformed the combination therapy involving CAR-T cells and the PD1 antibody, pembrolizumab [69,70].

In contrast, this study introduces an innovative strategy that employs the PD1/CD28 chimeric signaling receptors and synNotch pathways to modulate immune cell function. The PD1/CD28 CSR design converts the PD1 inhibitory signal into a CD28 activation signal in the NK cells. Simultaneously, synNotch receptors enable targeted IL-12 secretion using engineered NK cells, offering precise control over localized IL-12 delivery. This approach is particularly significant due to the limitations surrounding the systemic administration of IL-12 owing to severe side effects. This study builds upon this foundation by investigating the relationship between PDL1 expression on tumor cells, IL-12 production, and IFNγ secretion by the PD1-Syn-IL-12-NK cells in a co-culture media (Figure 5). A positive correlation between the PDL1 levels on tumor cells, IL-12 production by engineered NK cells, and increased IFNγ secretion highlighted the immunostimulatory potential of this construct against the PDL1-expressing tumor cells (Figure 5). A cytotoxicity assay further demonstrated the capacity of the PD1-Syn-IL-12-NK92 cells to augment the cytolytic activity of CAR-NK cells against PDL1-positive breast cancer target cells (Figure 6). This finding offers a promising avenue for developing immunotherapeutic strategies in breast cancer.

In addition to the activation signal triggered by the PD1/CD28 CSR binding to the PDL1+ cells, this study introduces an intriguing alternative approach. This approach addresses the compensatory effect of PD1 over-expression on activated T cells within the tumor microenvironment by competitively binding with PDL1 molecules on tumor cells. This competitive binding mechanism has the potential to hinder the function of the CAR-modified NK cells. CAR-NK cells have gained considerable attention recently due to their pivotal role in cancer immunosurveillance and their unique intrinsic features. These characteristics include their rapid identification and elimination of tumor cells, which can lead to graft-versus-tumor effects. Notably, CAR-NK cells share similarities with T cells, as they can be engineered to enhance their activity by incorporating signaling components and CARs targeting specific malignancy-related antigens. This dual approach holds promise for advancing cancer immunotherapy [71]. Preclinical research has provided evidence of the success of CAR-NK cells in targeting a range of tumor antigens [72,73].

## 5. Conclusions

In conclusion, this study underscores the promising potential of synNotch receptor-engineered NK cells in augmenting antitumor activity, with a specific focus on addressing the breast cancer cells characterized by elevated PDL1 expression. Our research highlights the precision achieved in delivering IL-12 directly to the tumor site, significantly improving antitumor responses while minimizing collateral damage. This controlled modulation of the immune microenvironment within the tumor represents a crucial pivotal advancement in cancer therapy and instils optimism regarding the development of more effective and targeted treatment strategies. These personalized approaches hold great promise for improving treatment outcomes not only in breast cancer but also in other challenging malignancies. This research paves the way for further investigation and development, advancing our pursuit of enhanced and more precise cancer treatments.

## Data Availability

The data presented in this study are available on request from the corresponding author. The data are not publicly available due to privacy.
